# Transformation of Silver Nanoparticles (AgNPs) during Lime Treatment of Wastewater Sludge and Their Impact on Soil Bacteria

**DOI:** 10.3390/nano11092330

**Published:** 2021-09-07

**Authors:** Zainab Abdulsada, Richard Kibbee, Juliska Princz, Maria DeRosa, Banu Örmeci

**Affiliations:** 1 Department of Civil and Environmental Engineering, Carleton University, Ottawa, ON K1S 5B6, Canada; zainab.abdulsada@carleton.ca (Z.A.); richard.kibbee@carleton.ca (R.K.); 2 Department of Environmental Engineering, University of Baghdad, Karrada, Al-Jadriya, Baghdad, Iraq; 3 Environment and Climate Change Canada, 335 River Road South, Ottawa, ON K1V 1C7, Canada; juliska.princz@canada.ca; 4 Department of Chemistry, Carleton University, Ottawa, ON K1S 5B6, Canada; Maria.DeRosa@carleton.ca

**Keywords:** silver nanoparticles, sludge, lime stabilization, land application of biosolids, bacterial phyla

## Abstract

This study investigated the impact of lime stabilization on the fate and transformation of AgNPs. It also evaluated the changes in the population and diversity of the five most relevant bacterial phyla in soil after applying lime-stabilized sludge containing AgNPs. The study was performed by spiking an environmentally relevant concentration of AgNPs (2 mg AgNPs/g TS) in sludge, applying lime stabilization to increase pH to above 12 for two hours, and applying lime-treated sludge to soil samples. Transmission electron microscopy (TEM) and energy-dispersive X-ray spectroscopy (EDS) were used to investigate the morphological and compositional changes of AgNPs during lime stabilization. After the application of lime stabilized sludge to the soil, soil samples were periodically analyzed for total genomic DNA and changes in bacterial phyla diversity using quantitative polymerase chain reaction (qPCR). The results showed that lime treatment effectively removed AgNPs from the aqueous phase, and AgNPs were deposited on the lime molecules. The results revealed that AgNPs did not significantly impact the presence and diversity of the assessed phyla in the soil. However, lime stabilized sludge with AgNPs affected the abundance of each phylum over time. No significant effects on the soil total organic carbon (TOC), heterotrophic plate count (HPC), and percentage of the live cells were observed.

## 1. Introduction

The nanoparticle industry is growing exponentially, and new nanomaterials and products are being introduced to consumers on an almost daily basis. AgNPs are commonly used as antimicrobial agents and incorporated in a wide range of merchandise and applications. Many products such as textiles, antimicrobial coatings, keyboards, wound dressings, and biomedical devices contain AgNPs that continually release a low level of silver ions [1]. AgNPs can easily bleed out of textiles in just a few washing cycles [2,3,4]. It also was reported that just one wash cycle could increase the total amount of silver released from 1% to 45%, depending on the type of fabric and the manufacturing methods [3]. Thus, a large portion of the AgNPs ends up in wastewater treatment plants [5,6]. In addition, studies have shown the toxicity of AgNPs on microorganisms that are necessary for wastewater treatment [7,8].

The sewer system is considered a primary source for the release of AgNPs into the environment. AgNPs are involved in a wide range of physical, chemical, and biological reactions after they are introduced into the sewer system and during wastewater and sludge treatment [9,10]. Although the dissolution of AgNPs has been observed, association with solids is reportedly the primary removal mechanism from wastewater [11,12]. More than 94% of AgNPs entering wastewater treatment plants are associated with sludge solids after treatment [12].

Treated sludge, also known as biosolids, is rich in nutrients essential for plant growth, including carbon, nitrogen, and phosphorous, and they are applied to improve soil properties such as texture and water holding capacity [13,14]. However, studies have shown that land application of biosolids can add harmful substances to soil [15]. Some studies also showed that the antimicrobial activity of AgNPs could have adverse effects on soil microorganisms and possibly affect the organisms necessary for plant growth [16,17].

Lime stabilization is a widely used sludge treatment method, as it is easy to apply, inexpensive, and beneficial for agriculture [13,18]. Lime stabilization works by raising sludge pH to 12 for at least two hours, which terminates microbial activity and allows small particles to coagulate into larger particles that can easily be separated from the aqueous phase. In addition to chemical stabilization, lime may also be used as a conditioning chemical during treatment to achieve coagulation and flocculation [19]. Lime is a harsh chemical that can impact the physical, chemical, and morphological characteristics of AgNPs in sludge and thereby change their impact on soil biological activity. It was reported that Ag_2_S was the dominant silver compound found after heat and lime treatment of sludge, with no Ag(0) observed [20]. Properties of AgNPs may be altered after interaction with sludge constituents or treatment chemicals. For example, the effect of the surface coating of AgNPs in soil matrices in terms of colloidal stability was annulled when AgNPs were applied with sewage sludge [21]. Thus, research is needed to better understand the effect of sludge treatment processes on AgNPs and whether AgNPs in sludge pose significant risks after land application. The goal of this study was to understand the fate and transformation of AgNPs during lime treatment of sludge at high pH values, as well as the impact of lime treated AgNPs on soil biological activity and health after simulated land application of sludge.

## 2. Materials and Methods

### 2.1. Sludge Characteristics

Anaerobically digested sludge was collected from mesophilic anaerobic digesters and obtained from the wastewater treatment plant in Ottawa, Canada. The plant receives about 390 ML/d of wastewater from residential, commercial, and industrial sources [22]. The chemical properties of the anaerobically digested sludge are provided in Table 1. After collection, sludge was stored at 4 °C until use.

### 2.2. AgNPs Characteristics

A stock suspension of pristine AgNPs in water with a purity of 99.9% was purchased from nanoComposix Inc. (San Diego, CA, USA). The properties of the AgNPs are provided in Table 2. The particle size distribution of AgNPs was examined and confirmed by TEM analysis. The AgNPs suspension stock solution was maintained at 4 °C until use.

### 2.3. Lime Treatment

Quicklime (CaO) with a molecular weight of 56.08 g/mol was purchased from Sigma-Aldrich. A lime slurry was prepared by mixing powdered lime with water at a concentration of 150 mg/mL (15%). The lime slurry was then mixed with anaerobically digested sludge at a concentration that maintained the pH above 12 for 2 h.

### 2.4. Soil Characteristics

Loamy topsoil was used in this study; it was free from additions such as compost or chemical fertilizer and was obtained from Artistic Landscape Designs Ltd. The soil was processed through a sieve with 2 mm mesh, air-dried and stored at 4 °C until use. Initial soil analysis included total organic carbon (TOC), total nitrogen (TN), phosphorus, pH, and cation exchange capacity (CEC) and are shown in Table 3.

### 2.5. Experimental Design

This study involved two stages. The first stage investigated the effect of lime treatment on the fate and behavior of AgNPs in sludge and the second stage investigated the effect of lime treated AgNPs on soil after simulated biosolids application. The concentration of AgNPs in sludge used in the first stage was more than twice as much compared to the second stage to enable the detection and visual observation of the AgNPs by TEM. Lime treatment was applied on two different test matrices; nanopure water and anaerobically digested sludge. Nanopure water was used as a control matrix to observe the interaction between AgNPs and lime in the absence of other contaminants. There were two test reactors for both nanopure water and sludge; one was the control, which had no lime treatment, and the other reactor was subjected to lime treatment. Each reactor contained 5 mL of water or sludge with two replicates each (experiment replicates). AgNPs were added to each reactor at a concentration of 0.1 mg/mL (corresponding to 5.64 ± 1.05 mg AgNPs/g sludge (dry weight) for the sludge reactor) and vortexed for 30 s. Lime stabilization was performed by adding lime to each reactor while continuously measuring the pH until a pH of 12 was reached and maintained for two hours, as shown in Supplementary Materials, Table S1. After lime treatment, water and sludge test reactor samples were prepared for TEM analysis by placing approximately 5 µL of the water and treated sludge on separate 3.05 mm, 200 mesh (grid hole size ≈ 97 nm) formvar/carbon-coated support copper grid film from Ted Pella Inc. Three sample replicates were obtained for each sample, and after preparation, the samples were analyzed using an FEI Tecnai G2 F20 TEM at the Nano Imaging Facility (NIF), Carleton University. In addition to morphological analyses, the elemental composition of the samples was analyzed using EDS coupled to TEM. This combination allows EDS to provide an elemental composition of an area that is only a few nanometers in diameter. The results are shown as a plot of the emitted X-rays versus their energy in keV; the different peaks correspond to specific elements.

The second stage of the study investigated the effect of AgNPs on the relative population of selected bacterial phyla in soil reactors after simulated land application. Test reactors were prepared in 250 mL clean glass beakers filled with 100 g dry soil. Each soil-filled reactor received sludge that was previously mixed with AgNPs and mixed thoroughly with a spatula. There were three reactors and two replicates for each reactor. There were two control reactors: control 1 received untreated anaerobically digested sludge without AgNPs, while control 2 received the same sludge previously mixed with AgNPs. The other reactor received sludge mixed with AgNPs and subjected to lime treatment. The final soil moisture content was 60% at the start of the experiment. The sludge/soil ratio was 0.01 g/g (dry weight), and it was selected based on the biosolids land application guidelines at a rate of 8 tons/ha every 5 years [23]. Additionally, other studies were considered in choosing the sludge application ratio [21,24,25]. The applied sludge was previously spiked with AgNPs suspension to obtain a final concentration of 2 mg AgNPs/g sludge (dry weight) corresponding to 20 mg AgNPs/kg soil when sludge was combined with soil. The AgNPs concentration used is an environmentally relevant concentration and represents the higher range published values of the recorded and predicted concentrations in wastewater sludge [5,6,26,27]. The reactors were incubated with day/night cycles of 8/16 h at a constant temperature of 22 ± 1 °C. This study was carried out for 105 d, with soil chemical and biological analyses conducted every 15 d, with the exception of moisture content, which was monitored every three days. Chemical analyses included cation exchange capacity (CEC), pH, total solids (TS), moisture content (MC), and total organic carbon (TOC). Biological analyses consisted of heterotrophic plate count (HPC), live/dead bacterial viability staining, and DNA analyses using quantitative polymerase chain reaction (qPCR). Similar concentrations of AgNPs were used in previous studies [7,28].

### 2.6. Chemical Analysis

The chemical analysis included the initial analysis of soil and sludge samples in addition to the analyses that were carried out every 15 d from the start of the study to its completion. All chemical analyses were completed in triplicate following Standard Methods [29]. The soil was analyzed for CEC at the beginning of the experiment using the standard method employed by the Natural Resources Conservation Service [30]. The analysis method involved the use of 1 M ammonium acetate (NH_4_OAc) at pH 7 (neutral NH_4_OAc). Soil samples were prepared for pH analysis by first suspending 5 g of soil in 5 mL of water (1 g:1 mL ratio) followed by a 30 s vortex of the suspension. The pH was measured using the Orion Star TM A326 pH/DO portable meter. The percentages of TS and MC were determined by drying 5 g of the soil sample in an oven at 105°C for 24 h. TS% represented the sample weight after drying divided by sample weight before drying and multiplying by 100. MC was calculated by taking the difference in the sample weight before and after drying divided by the weight of the dried sample and multiplying by 100%. MC was monitored every 2–3 days and maintained at 45% by the addition of sterile distilled water. TOC analysis was conducted using TOC-VCPH/CPN and TOC-control V software. Each TOC measurement run included one blank and a set of standard solution concentrations that were used to construct a calibration curve. Three replicates were taken for each TOC measurement. Soil samples were prepared by suspending 1 g of soil in 10 mL of CaCl_2_ solution (1:10 *w*/*v*, 0.01 M). The suspension was put in a shaker for 2 h and centrifuged at 1800× *g* for 12 min. The supernatant was then used for the TOC analysis; these results represent soluble TOC.

### 2.7. Microbiological Analyses

#### 2.7.1. Heterotrophic Bacteria

The viability of heterotrophic bacteria in the soil samples was investigated every 15 days using the spread plate method. Tryptic soy agar (TSA) was used as the growth medium. TSA was prepared by adding 30 g of the powdered medium to 1 L of distilled water, dissolved by boiling and autoclave sterilized. After the TSA had cooled to approximately 50 °C, a volume of 22 mL was then poured into each Petri plate and stored at 4 °C for a maximum of one week. Soil samples were prepared by suspending 0.1 g of the soil sample in 0.9 mL PBS (with 0.1% Tween80) and vortexed for 30 s. Serial dilutions were made, and three replicates of 0.1 mL of 10^−4^, 10^−5^, and 10^−6^ dilutions were inoculated onto the TSA plates and incubated in the dark at 34 ± 1 °C. Colony-forming units (CFU) were calculated after a 24 h incubation time.

#### 2.7.2. Live/Dead Bacterial Viability Staining Assay

The distribution of live/dead bacterial cells in the soil reactors was investigated by staining with fluorescent dyes and observed with a Nikon Eclipse Ti-E Microscope. The soil samples were stained with a BacLight staining reagent kit that was prepared by dissolving SYTO 9 stain and propidium iodide stain in 5 mL sterilized deionized water. The soil samples were prepared for live/dead analysis by suspending 0.1 g of soil in a 0.9 mL of PBS solution with 0.1% Tween80. The mixture was then vortexed for 30 s at high speed and allowed to settle for 30 min thereafter. A known volume of the supernatant was mixed with an equal amount of the staining reagent, and the mixture was incubated in the dark for 15 min. Then, a 17 µL of the mixture was applied to a standard microscope slide and covered with a 22 mm square coverslip. The samples were then ready for observation under the fluorescence microscope, where the ratio of live/dead bacterial cells could be quantified. Three tests were undertaken for each soil sample, and three random fields of views (FOV) were chosen on each slide where images were taken. The images were taken at each FOV using 3 UV filters, GFP-1, Cy-3, and GFPHQ to capture live, dead, and a combination of live and dead microbial cells, respectively. The three sets of images were assessed, and the most representative was chosen to report, and the live cell percentage was estimated by dividing the number of live cells by the total number of cells in the image.

#### 2.7.3. Bacterial Population Analysis

##### Bacterial Phyla

The five most prevalent bacterial phyla found in soil and sludge were selected for investigations: Acidobacteria, Actinobacteria, Bacteroidetes, Firmicutes, and Proteobacteria. Each soil reactor was analyzed for the presence and relative population of each phylum every 15 d throughout the 105-d experiment. Three replicate DNA extractions were taken for each soil reactor using the PowerSoil DNA Isolation Kit from MO BIO Laboratories Inc. (QIAGEN Online Shop). The concentration of extracted DNA was measured using the Qubit^®^ 2.0 Fluorometer with the dsDNA HS Assay Kits (Molecular Probes, Life technology). The extracted DNA was then used as template DNA for the phylum-specific qPCR protocols. Each phylum was amplified individually using phylum-specific qPCR primer sets.

##### Primers

Primers were purchased from Life Technologies (Burlington, ON, Canada). The primers were selected based on recently published studies. Primer references and properties are shown in Table 4.

##### qPCR Protocols and Annealing Temperature Optimization

Phylum analysis was conducted using individual qPCR protocols for each sludge/soil mixture every 15 d throughout the experiment. In each qPCR run, there were three replicate qPCR reactions per genomic DNA sample for each phylum with three non-template controls (NTC) in each run. Reactions were performed using CFX96 Touch Real-Time PCR Detection System (BioRad) and analyzed with the CFX Manager Software. The qPCR protocol was the same for all phyla except for the annealing temperature, which was different for each phylum. The qPCR protocol consisted of 95 °C for 5 min as an initial denaturation step, followed by 40 cycles of a 95 °C denaturation for 10 s, annealing for 20 s (Table 4), and an extension of 72 °C for 15 s, and a melt curve analysis from 65 to 95 °C with a 0.5 °C increase every 5 s. Every qPCR reaction consisted of 12.5 μL qPCR buffer, 0.5 μL (200 nM) forward primer, 0.5 μL (200 nM) reverse primer, DNA template equilibrated to 30 ng concentration, and water to 25 μL as the final reaction volume. The qPCR reaction/buffer solution was SsoFast EvaGreen Supermix (purchased from Bio-Rad Laboratories Ltd., Canada) and contained dNTPs, Sso7d fusion polymerase, MgCl_2_, EvaGreen dye, and stabilizers. Preliminary experiments were conducted to optimize the annealing temperature for each phylum protocol. The annealing temperature that maximized amplification was determined with extracted soil DNA template in a thermal gradient qPCR reaction with a different annealing temperature range for each phylum.

##### qPCR Data Analysis

Universal 16S ribosomal RNA primers were used to amplify an ~180 bp sequence which was used to calculate the baseline 16S gene copy number and was compared to each of the five phyla-specific calculated gene copy numbers. The data generated relative population trends for each phylum over time. The data analysis was based on three main fundamental assumptions:

1. Cq is equivalent to 3.33 log10 (target amplicon concentration).

Many studies have used a control curve to relate the number (concentration) of DNA in the qPCR product to the corresponding Cq. A study has mentioned that constructing a control curve with Log10 of DNA number versus Cq (resulted from the qPCR run of serial concentrations of DNA) will be linear with a slope of 3.33 when the qPCR efficiency is 100% [35].

2. Every bacterium has 1~ fg of DNA.

The concentration of the DNA template used in the qPCR reaction was 30 ng. The qPCR analysis in this study relies on a relative estimation of the bacterial population. If it is assumed that every bacterium has 1~ fg of DNA, then 30 ng of DNA equal to 3 × 10^7^ bacteria. This is just a relative estimation because the soil extracted DNA belongs to many other living things besides bacteria, such as plants and eukaryotes.

3. Every phylum has a specific copy number of 16S rRNA.

Based on a study by Větrovský and Baldrian (2013), each phylum has a specific copy number of 16S rRNA, as illustrated in Table 5 [36].

### 2.8. Statistical Analyses

Most results are reported as the average of three replicates ± standard deviation. Comparisons and significant differences between different soil reactors over time (control 1 versus control 2 and control 2 versus the lime reactor) were performed using a one-way analysis of variation (ANOVA) at 95% confidence level. Significant differences are indicated by a *p*-value < 0.05. If the ANOVA-test indicated a significant effect, a *t*-test was performed between each of the two data sets. In addition, the coefficient of variance (CV) was estimated for each data set, and significant differences are those with a CV >0.05 (i.e., the level of confidence ≥95%).

## 3. Results and Discussion

### 3.1. Characterization of AgNPs

**Figure 1:**
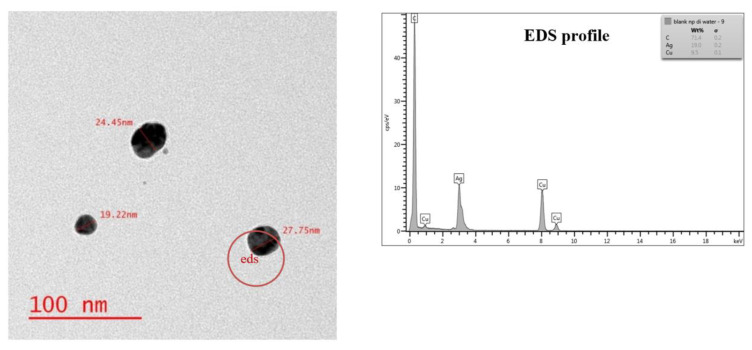
TEM image of AgNPs suspended in water with its EDS profile.

The AgNPs suspension was examined for particle size and morphology using TEM analysis after dilution of the stock solution with nanopure water; the TEM images revealed that the AgNPs suspension was extremely pure. The images were examined with EDS to determine the elemental composition of the sample, and other than carbon and copper, which were components of the supportive grid, only silver was found (Figure 1). During TEM imaging, the AgNPs were well dispersed within the water, and agglomeration or aggregation of particles was not detected. This is likely due to the PVP coating that helped the particles resist clustering and deposition. PVP coated AgNPs were employed in this study, since they are very widely used in the industry and are present in sludge.

### 3.2. Lime Stabilization

**Figure 2:**
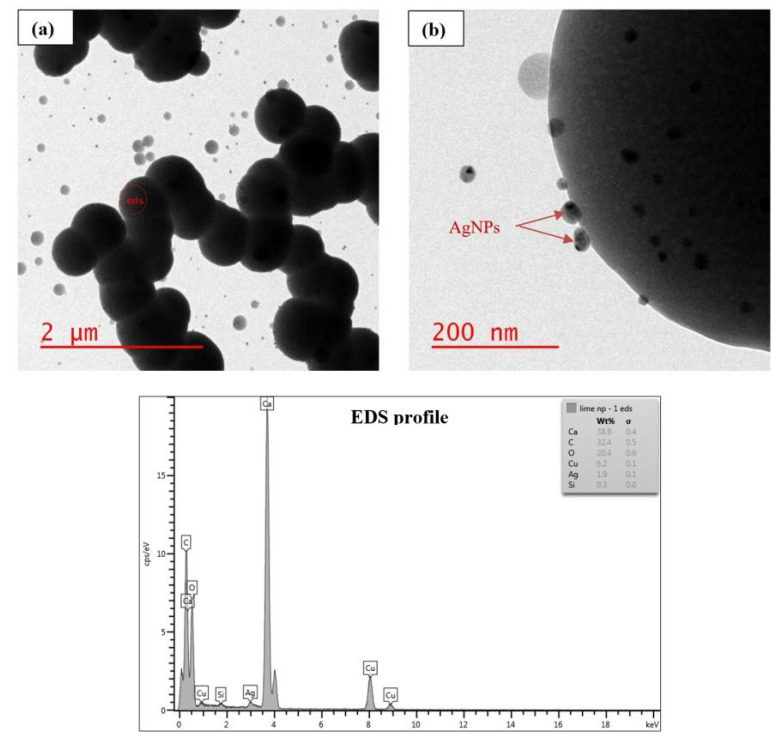
TEM images show lime treatment effect on AgNPs suspended in nanopure water, with EDS profile. (**a**,**b**) refer to different scales.

**Figure 3:**
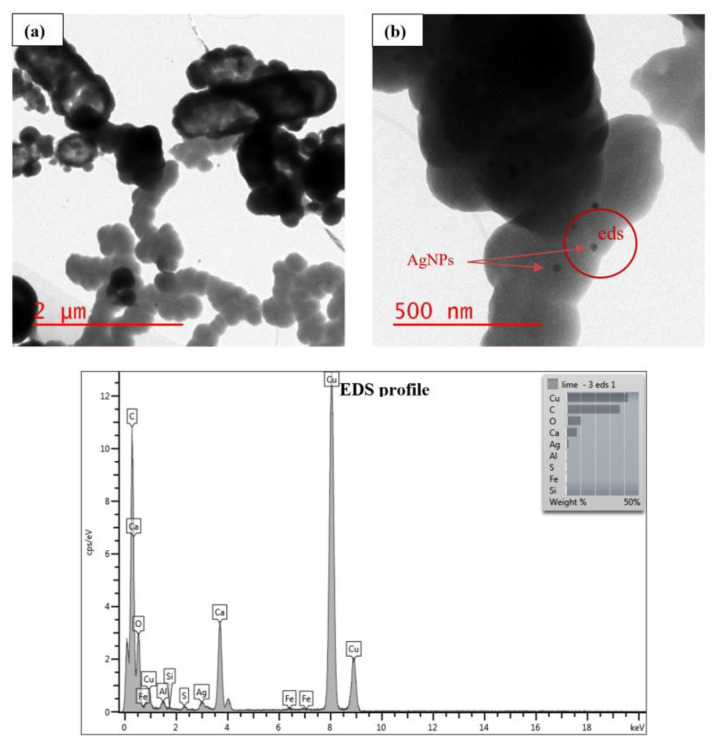
TEM images show lime treatment effect on AgNPs suspended in sludge, with EDS profile. (**a**,**b**) refer to different scales.

During the first stage of the study, AgNPs were added to sludge at a concentration of 0.1 mg AgNPs/mL sludge, then lime treatment was applied. The treated sludge was then examined for morphological and structural changes of AgNPs using TEM. The visual observation revealed the formation of larger, less mobile particle clusters due to coagulation caused by lime particles. TEM images showed that lime removed AgNPs effectively from the aqueous phase and that AgNPs were deposited to the surface of lime molecules, as shown in Figure 2 and Figure 3. The images also showed that AgNPs were still in the nanoparticle form after lime treatment. More AgNPs were attached to the surface of lime molecules in water (Figure 2) than in sludge (Figure 3). This is likely because there were more competing reactions among AgNPs, lime, and sludge constituents, thereby decreasing the availability of AgNPs for surface attachment. Using EDS, several elements were detected, including Ag and Ca in water (Figure 2), and Ag, Ca, Fe, Al, and S in sludge (Figure 3). Lime works by raising the pH of the sludge and increasing its porosity. Lime also causes hydrogen sulfide to revert to sulfide and bisulfate ions, which are not volatile at elevated pH levels and hence reduce sludge odor [19]. A study showed that Ag_2_S is the dominant silver compound found after sludge undergoes lime and heat treatment [20].

### 3.3. Simulated Land Application of Lime Stabilized AgNPs

#### 3.3.1. TOC and pH

**Figure 4:**
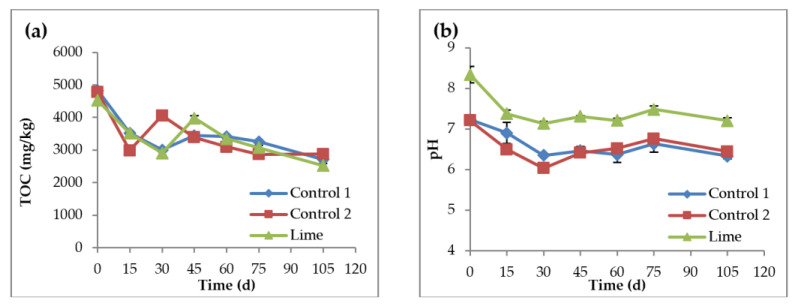
Total organic carbon concentration (**a**) and pH (**b**), over time. Control 1 is the soil reactor that received untreated sludge with no nanoparticles, control 2 is the soil reactor that received untreated sludge with 2 mg AgNPs/g TS sludge, and lime is the soil reactor that received lime treated sludge with 2 mg AgNPs/g TS sludge.

After land application of lime stabilized sludge, TOC decreased over 105 days at a similar rate in all soil reactors, as shown in Figure 4a. TOC of the two controls and lime soil reactors were similar, with no significant differences observed. *t*-test, *p*-values between controls 1 and 2 and between control 2 and the lime reactors were 0.96 and 0.94, respectively. TOC for all reactors decreased significantly during the first month, with an overall reduction of 44%, 40%, and 44.4% for controls 1 and 2, and lime reactors, respectively. Organic soil carbon plays a major role in overall soil health, as it is the food source for soil microorganisms and helps fixate soil nutrients such as nitrogen, phosphorus, and sulfur. Several factors can affect the TOC levels in soil, including pH, temperature, soil aeration, and microbial population of the soil. The observed decrease in TOC was likely due to the acclimatization time, as well as the decomposition and consumption of organic matter by microorganisms. Microorganisms compete with each other to survive in a limited carbon environment, and no carbon source was added during the experiment.

Soil pH, a parameter with a major influence on the availability of nutrients such as nitrogen and phosphorus in the soil, is essential for soil microbial population and diversity, and several studies have shown the impact of pH on AgNPs [37]. The soil pH levels were not affected by the presence of AgNPs and decreased during the first 15 days for all soil reactors, as seen in Figure 4b. This is normal and due to chemical reactions and acclimation to the new environment by the microorganisms. The pH readings were stable over the remaining period, and after day 15, the pH control reactor dropped from neutral (an approximate pH value of 7) at the beginning of the experiment to somewhat acidic at the end. The pH of the lime reactor was alkaline (pH = 8.3) on day 0 and decreased to near neutral (pH = 7.2) at the end of the experiment.

#### 3.3.2. Impact of AgNPs on Heterotrophic Bacteria and Cell Viability

**Figure 5:**
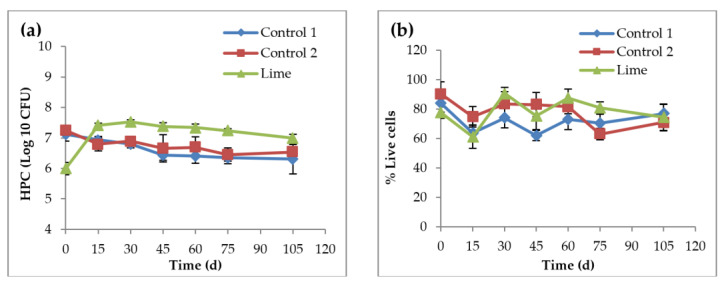
HPC, represented by CFU (**a**) and the percentage of live cells in soil reactors (**b**) over time. Control 1 is the soil reactor that received untreated sludge with no nanoparticles, control 2 is the soil reactor that received untreated sludge with 2 mg AgNPs/g TS sludge, and lime is the soil reactor that received lime treated sludge with 2 mg AgNPs/g TS sludge.

Under lime treatment, heterotrophic bacteria showed slightly different trends over time compared to controls 1 and 2. On day 0, as seen in Figure 5a, HPC for a lime soil reactor was lower by ~1 Log10 CFU/mL, most likely due to the effect of high pH. However, CFU under lime treatment showed the highest increase over time (16.6% increase), compared to controls 1 (11.4% decrease) and 2 (9.8% decrease), as shown in Figure 5a. This is likely due to the solubilization of substrates after lime treatment, which makes them readily available for microorganisms. The *t*-test showed no significant difference between controls 1 and 2 (*p*-value ≈ 0.58). Thus, the presence of AgNPs at this concentration had no significant effect on the heterotrophic bacteria in the soil. This finding is supported by other studies that showed no effect of AgNPs on heterotrophic bacteria [7], even though it was reported that lime seemed to enhance CFU. HPC results showed significant differences in the number of heterotrophic colonies between control 2 and the lime reactor (*p*-value ≈ 0.02), which indicates that lime treatment of sludge significantly impacted the heterotrophic bacteria in the soil.

In addition to the enumeration of the heterotrophic bacteria, live/dead viability staining was also evaluated using two fluorescent dyes, CYTO 9 and propidium iodide. Bacterial cells with intact membranes are stained green, while those with damaged membranes are stained red. The percentage of live cells for both control reactors fluctuated over time, and they followed similar trends (increasing mode), and ANOVA analysis showed no significant differences between the controls. However, as shown in Figure 5b there were small increases in the percentage of live cells in all the soil reactors, indicating that neither the AgNPs nor the sludge lime treatment caused significant toxicity to soil bacteria at the concentrations applied. The bacterial live/dead staining images are presented in the Supplementary Materials, Figures S2–S4.

#### 3.3.3. Impact of AgNPs on Selected Soil Microorganisms

After extraction of the genomic DNA from the soil reactors, genomic was analyzed for the presence and concentration of five selected bacterial phyla using qPCR. DNA concentrations measured in the reactors over time are shown in Supplementary Materials, Figure S1. The Cq (quantitation cycle) values from the qPCR results were converted to calculated CFU equivalents (CCE) based on the assumptions discussed in the material and methods. CCE was used to estimate the mean abundance of each phylum in all reactors. Of the five phyla, Acidobacteria was the most highly abundant in all soil reactors, with 29% relative abundance, Proteobacteria was the next, with 26% abundance, and Bacteroidetes had 3% relative abundance, which was the least, while Firmicutes and Actinobacteria, both, had 21% relative abundance, and, thus, were similar in terms of having medium abundance compared to the other assessed phyla. These findings correspond with those of other researchers who have studied soil bacterial diversity and abundance [31,38].

All the studied phyla showed similar Cq values in controls 1 and 2 throughout the experiment with very little variance in their CCEs. *t*-test *p*-values were 0.04, 0.6, 0.52, 0.43 and 0.84 for Proteobacteria, Acidobacteria, Actinobacteria, Bacteroidetes and Firmicutes, respectively. The phyla relative abundance for the controls throughout the experiment was very similar, with variations between them of less than one Log10 CCE. Thus, AgNPs at a concentration of 20 mg AgNPs/g soil had minimal impact on the diversity and presence of these five phyla in soil.

**Figure 6:**
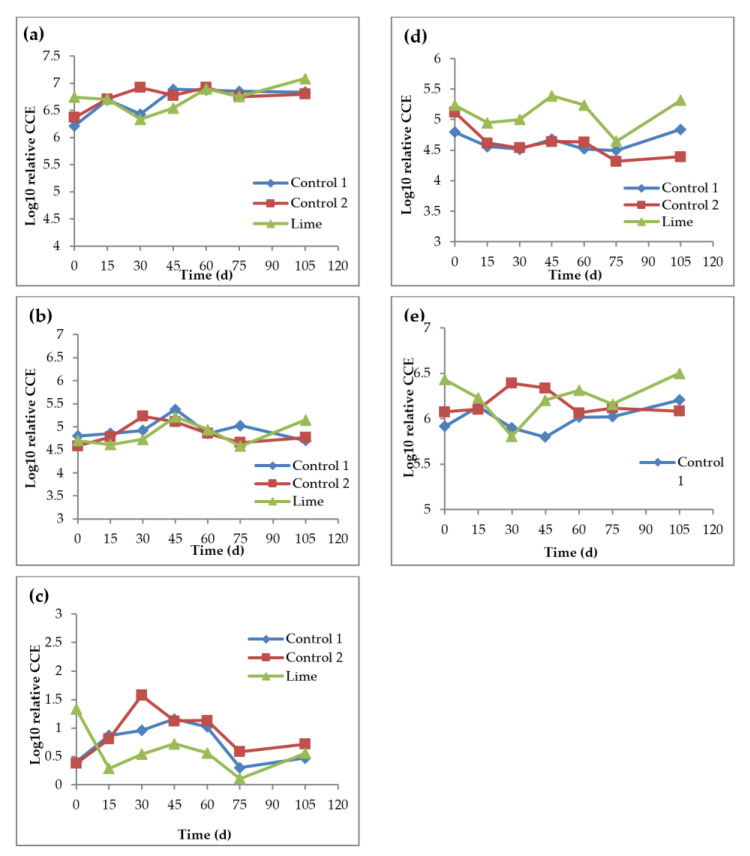
Relative change in CCE for Acidobacteria (**a**), Actinobacteria (**b**), Bacteroidetes (**c**), Firmicutes (**d**) and Proteobacteria (**e**). Control 1 is the soil reactor that received untreated sludge with no nanoparticles, control 2 is the soil reactor that received untreated sludge with 2 mg AgNPs/g TS sludge, and lime is the soil reactor that received lime treated sludge with 2 mg AgNPs/g TS sludge.

Assessment of phyla abundance in the soil reactors that received lime-treated sludge showed a higher degree of variation in Cq values (leading to dissimilar CCE profiles) compared to the controls (Figure 6). Under lime treatment, all studied phyla showed an increase in abundance except Bacteroidetes.

The CCE trends over time for Acidobacteria were similar in control 2 and lime reactors, and ANOVA showed no major differences in the CCE of Acidobacteria in all soil reactors (*p*-value >0.05). This indicates that Acidobacteria was not adversely affected by the presence of AgNPs or the sludge lime treatment; CCE increased by 10%, 6% and 5% for control 1, control 2 and lime soil reactors, respectively. This is likely due to the potential of Acidobacteria to tolerate various pollutants and heavy metals [39,40,41]. Acidobacteria is also responsible for several beneficial soil fertility functions, including nitrate and nitrite reduction [42].

Actinobacteria also showed similar CCE trends over time for control 2 and lime reactors, as seen in Figure 6. Analysis with ANOVA showed no major differences (*p*-value = 0.95) between control 2 and the lime reactor in relative CCE trends. However, lime treatments seemed to promote Actinobacteria abundance with a 9% increase in CCE throughout the experiment. Actinobacteria is one of the most abundant and important bacterial phyla in soil and helps with carbon degradation, including cellulose, CO_2_ fixation, nitrogen fixation, and phosphorus uptake [43]. Thus, Actinobacteria is an important factor to consider when studying the impact of nanoparticles on the soil. The relative CCE of Bacteroidetes also showed no significant differences between control 2 and the lime reactors, and ANOVA results showed a *p*-value of 0.16. However, Bacteroidetes CCE was virtually unchanged in controls 1 and 2, and it decreased by 6% under the lime treatment. Bacteroidetes have several important roles in soil, including solubilization of minerals such as phosphate, production of siderophores, and synthesis of growth-stimulating phytohormones [44]. The results of the relative CCE of Firmicutes showed a significant difference between control 2 and the lime reactor (*p*-value = 0.0034), although this was due to the variation in the CCE at the start of the experiment, where the lime soil reactor had a higher CCE value, likely due to the effect of lime.

Firmicutes is an important soil bacterial phylum, as it includes a number of genera, such as *Bacillus* and *Paenibacillus* that are key to soil fertility, including atmospheric nitrogen-fixation, solubilization of minerals, suppression of plant pathogens, production of siderophores, synthesis of growth-stimulating phytohormones and bioremediation [45,46,47]. Thus, the results showed no significant impact of AgNPs on the abundance of Firmicutes, but sludge lime treatment enhanced their abundance.

The trends over time of Proteobacteria CCE exhibited no major differences between control 2 and the lime reactor (*p*-value = 0.5). There was no significant impact of AgNPs at 20 mg/kg soil, or with sludge lime treatment, on the abundance of Proteobacteria in the soil. Proteobacteria are common in soil and play an important role in soil fertility functions, including carbon degradation such as aromatic compounds, CO_2_ fixation, nitrogen fixation, oxidation of iron, sulfur, and methane, phosphorus uptake and suppression of plant pathogens [43,48]. Given the diversity of its important roles in soil, the inclusion of Proteobacteria is required when assessing the potential toxicity of nanoparticles.

The stability, transformation, and subsequent toxicity of AgNPs after release into the environment depend on several factors, such as the complexity of the media, the forms of the nanoparticles, surface chemistry, concentration, and exposure time. In this study, interaction with sludge constituents and lime affected the physical and chemical characteristics of AgNPs. For example, organic matter present in sludge and soil could coat the surface of AgNPs, thereby stabilizing them and reducing their aggregation and toxicity [49,50]. Moreover, lime treatment affects the chemical and physical characteristics of sludge through several reactions, such as hydrolysis, saponification, and acid neutralization, which can affect the surface chemistry of AgNPs and alter their behavior and interaction. Soil bacteria are the microbial community responsible for several soil ecosystem functions, including recycling of organic matter and nutrients, degradation of toxins such as heavy metals, and suppression of pathogens [51,52,53]. Several studies have reported the negative impact of AgNPs on soil microbial activities [54], and it is important to investigate the effect of lime-treated AgNPs.

Overall, this study found no significant variations between the controls with and without AgNPs for the five assessed phyla. These findings were supported by no significant differences in HPC and the percentage of live cells. This indicates that AgNPs at a concentration of 20 mg AgNPs/kg soil did not show a significant impact on soil biological activities based on the assessed parameters. This could be due to several factors, such as the AgNPs concentration used in this study, which was environmentally relevant but might not have been high enough to exhibit toxicity. Another factor could be the interaction of AgNPs with sludge constituents that results in physicochemical transformations before soil application. This could alter AgNPs behavior and affect bioavailability and toxicity and could include mechanisms such as reduction, oxidation, aggregation, and dissolution [11,24,55,56,57]. For example, AgNPs interact strongly with sulfur and produce silver sulfide (Ag_2_S) when they are discharged into the sewer system and during wastewater treatment [56,57]. The interaction of AgNPs with sulfur and the subsequent production of silver sulfide results in a significant reduction in the toxicity of AgNPs due to the lower solubility of silver sulfide and limits potential short-term environmental impact [58].

## 4. Conclusions

This study investigated how lime stabilization affects the fate and transformation of AgNPs, and its impact on soil bacterial activity and health after simulated land application of the lime stabilized sludge. Lime treatment effectively removed AgNPs from the liquid phase and concentrated them in sludge solids. AgNPs were well incorporated in lime and were also deposited on the surface of lime molecules. The presence of AgNPs at 2 mg AgNPs/g TS of sludge (20 mg AgNPs/kg soil) did not have a significant impact on the population or abundance of Acidobacteria, Actinobacteria, Bacteroidetes, Firmicute, and Proteobacteria, which are important for soil health. Furthermore, no significant effects on the soil TOC, HPC, and the percentage of the live cells were observed.

## Figures and Tables

**Table 1:** Anaerobically digested sludge characteristics.

TP (Total Phosphorus)	TN (Total Nitrogen)	TS (Total Solids)	TVS (Total Volatile Solids)	pH
122 ± 8 mg/L	1043.3 ± 33 mg/L	17,720 ± 95 mg/L	9803.3 ± 45 mg/L	7.4 ± 0.1

Values are the mean of three replicate measurements ± standard deviation.

**Table 2:** AgNPs characteristics.

Diameter (TEM)	Coefficient of Variation	Surface Area (TEM)	Particle (Concentration)	Hydrodynamic Diameter	Zeta Potential	pH of the Solution	Particle Surface Coating	Solvent
23.1 ± 6.9 nm	29.8%	21.5 m^2^/g	7.2 × 10^13^ particles/mL	49.6 nm	−30.6 mV	6.3	Polyvinylpyrrolidone (PVP)	Milli-Q water

**Table 3:** Soil characteristics.

TOC	TN	TP	pH	CEC
4248 ± 12 mg/kg	184 ± 7 mg/kg	13 ± 2 mg/kg	6.6 ± 0.7	30.8 ± 2 meq/100 g

Values are the mean of three replicate measurements ± standard deviation.

**Table 4:** Phyla primer pairs used in qPCR assay.

Phylum	Amplicon Size (bp)	Primer Name	Primer Sequence (5′–> 3′)	Annealing Temperature (°C)	References
Acidobacteria	500	fAcid31	GAT CCT GGC TCA GAA TC	55.9	[31]
		rEub518	ATT ACC GCG GCT GG		
Actinobacteria	166	fActi	GRD ACY CCG GGG TYA ACT	57.2	[32]
		rActi	TCW GCG ATT ACT AGC GAC		
Bacteroidetes	181	fBdet	GCA CGG GTG MGT AAC RCG TAC CCT	61	[32]
		rBdet	GTR TCT CAG TDC CAR TGT GGG		
Firmicutes	156	fFirm	CAG TAG GGA ATC TTC	55.3	[32]
		rFirm	ACC TAC GTA TTA CCG CGG		
Proteobacteria	140	767fProt	AAG CGT GGG GAG CAA ACA	54.8	[33]
		907rProt	CCG TCA ATT CMT TTR AGT TT		
16S (any bacteria)	180	338F	ACT CCT ACG GGA GGC AGC AG	61.9	[34]
		518R	ATT ACC GCG GCT GG		

**Table 5:** Copy number of 16S rRNA in each bacterial genome [36].

Phylum	16S rRNA/Genome	Mean 16S rRNA/Genome
Acidobacteria	1	1
Actinobacteria	3.3 ± 1.7	3.3
Bacteroidetes	3.5 ± 1.5	3.5
Firmicutes	5.8 ± 2.8	5.8
Proteobacteria	3.96 ± 1.7	4.0

## Data Availability

Any further data can be provided upon personal request.

## Supplementary Materials

The following are available online at https://www.mdpi.com/article/10.3390/nano11092330/s1. Table S1. Lime doses and corresponding pH levels. Figure S1. DNA concentration for the controls and lime reactors over time. Figure S2. live/dead images of control 1 (the soil reactor that received untreated sludge with no nanoparticles). Figure S3. live/dead images of control 2 (the soil reactor that received untreated sludge with 2 mg AgNPs/g TS sludge). Figure S4. live/dead images of lime reactor (the soil reactor that received lime treated sludge with 2 mg AgNPs/g TS sludge.
