# Supplementary material for: Transformation of Silver Nanoparticles (AgNPs) during Lime Treatment of Wastewater Sludge and Their Impact on Soil Bacteria
Source: Nanomaterials (Basel). 2021 Sep 7;11(9):2330. doi: 10.3390/nano11092330 (PMC8465233; doi:10.3390/nano11092330)
Supplement: Supplementary file 1 [file nanomaterials-11-02330-s001.zip › nanomaterials-1333431-supplementary.pdf]

Supplementary Material

Transformation of Silver Nanoparticles (AgNPs) During Lime Treatment of Wastewater Sludge and Their Impact on Soil Bacteria

Zainab Abdulsada [1,2], Richard Kibbee [1], Juliska Princz [3], Maria DeRosa [4] and Banu Örmeci [1,*]

1 Department of Civil and Environmental Engineering, Carleton University, Ottawa, ON K1S 5B6, Canada; zainab.abdulsada@carleton.ca (Z.A.); richard.kibbee@carleton.ca (R.K.)
2 Department of Environmental Engineering, University of Baghdad, Karrada, Al-Jadriya, Baghdad, Iraq
3 Environment and Climate Change Canada, 335 River Road South, Ottawa, ON K1V 1C7, Canada; juliska.princz@canada.ca
4 Department of Chemistry, Carleton University, Ottawa, ON K1S 5B6, Canada; Maria.DeRosa@carleton.ca
* Correspondence: banu.ormeci@carleton.ca; Tel.: +1-613-520-2600 (ext.4144)

Table S1. Lime doses and corresponding pH levels.

Lime/TS of sludge (g/g)	pH
0.1	10.2
0.15	10.94
0.27	12.08
0.3	12.11

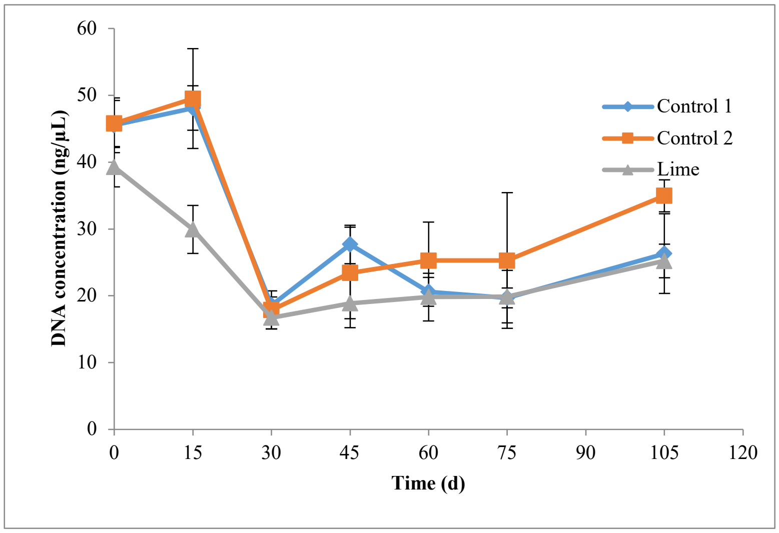

Figure S1. DNA concentration for the controls and lime reactors over time.

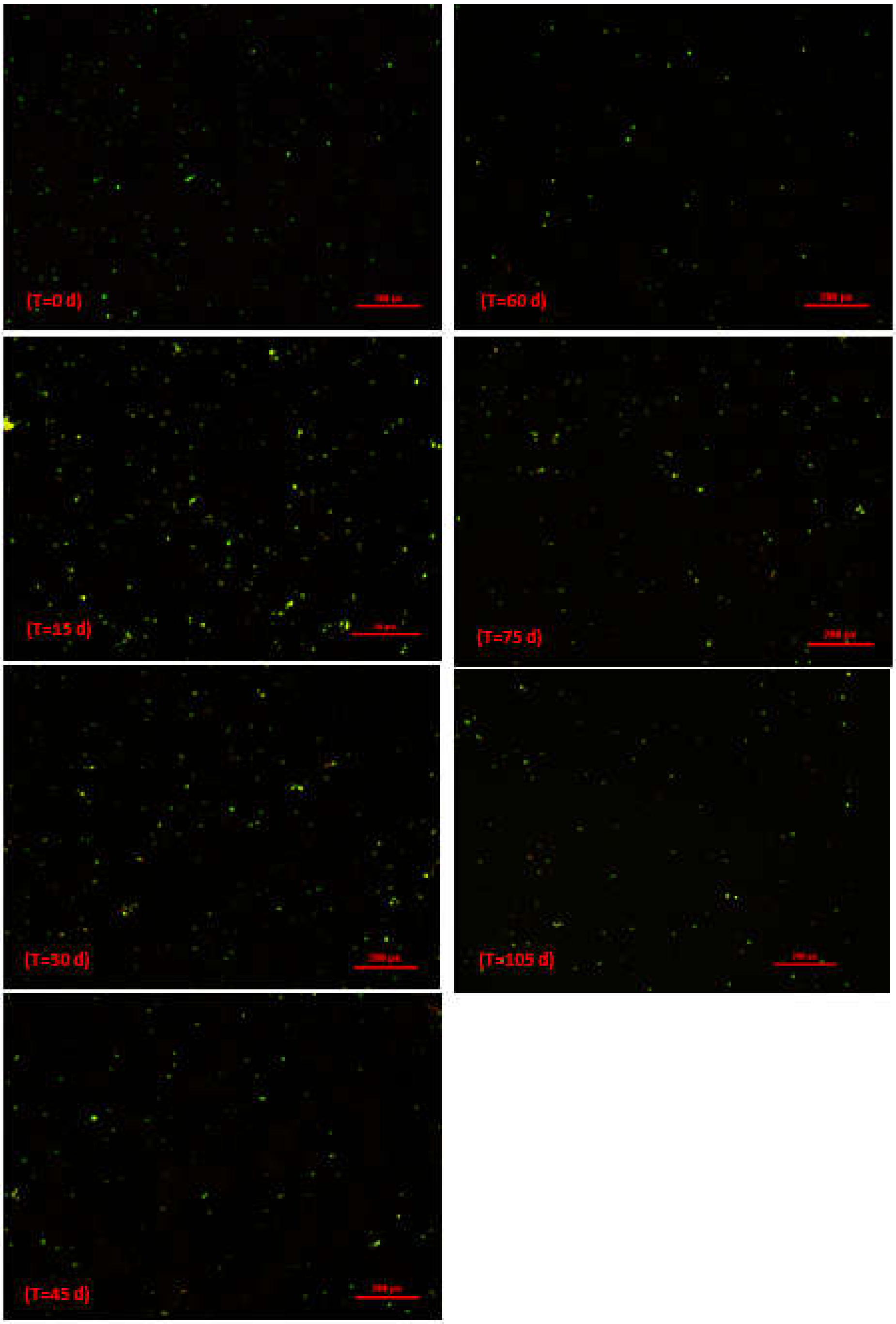

Figure S2. Live/Dead images of control 1 (the soil reactor that received untreated sludge with no nanoparticles).

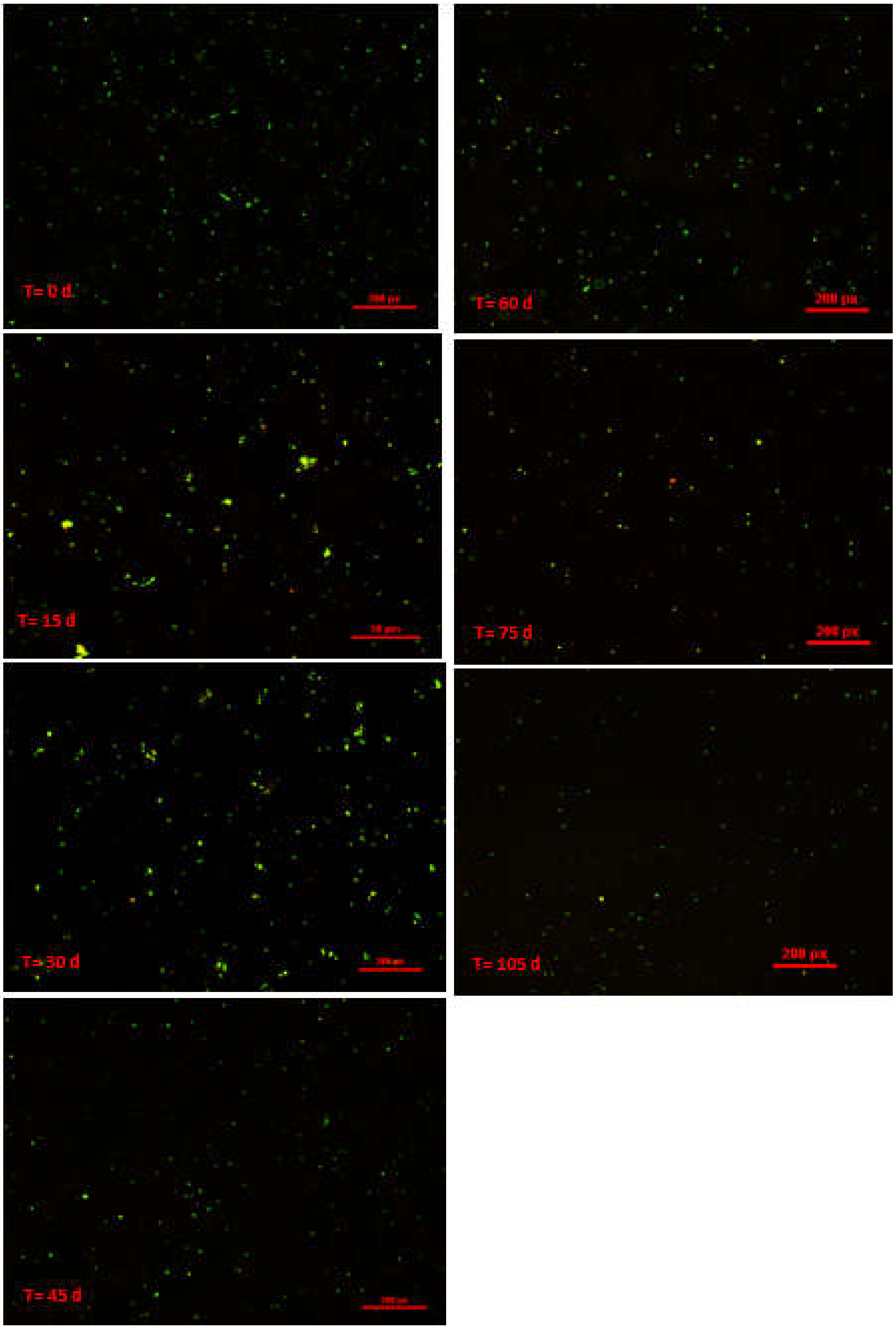

Figure S3. Live/Dead images of control 2 (the soil reactor that received untreated sludge with 2 mg AgNPs/g TS sludge).

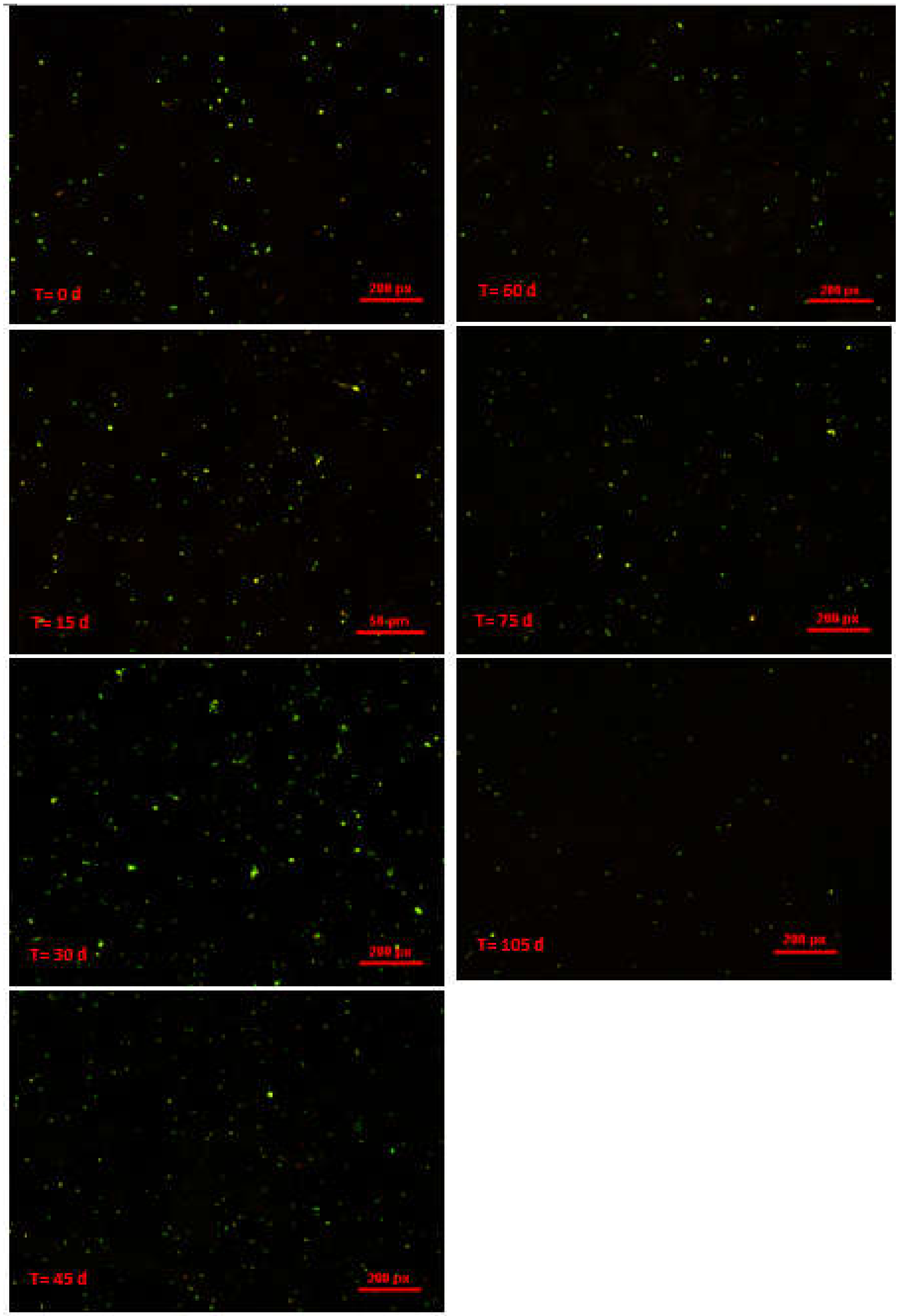

Figure S4. Live/Dead images of lime reactor (the soil reactor that received lime treated sludge with 2 mg AgNPs/g TS sludge.
